# HOW DOES AWARENESS OF SOCIAL SERVICES PLAY A ROLE IN THE RELATIONSHIP BETWEEN RELATIONAL RESOURCES AND THE AIP?

**DOI:** 10.1093/geroni/igae098.4208

**Published:** 2024-12-31

**Authors:** Junmin Park, Yeonjung Lee

**Affiliations:** Chung-Ang University, Seoul, Republic of Korea; Chung-Ang University, Seoul, Republic of Korea

## Abstract

The process of ‘Aging in Place (AIP)’ encompasses a variety of declines in physical capabilities, adaptability to environmental changes, and the breadth of interpersonal relationships. The AIP is not just about staying in the community; it also involves the availability of requisite resources to address the needs that emerge throughout the AIP journey. In this context, relational resources such as an individual’s social capital, may serve as a significant asset. Nonetheless, the availability of relational resources is not uniform among older adults, and it might be risky to solely rely on relational resources, potentially resulting in an excessive burden on the enhancement of AIP sustainability. Instead, social services can act as supportive resources. The purpose of this study is to examine the moderating role of perceived awareness of social services in the relationship between relational resources and the intention to AIP. Logistic regression was conducted using data from the 2020 National Survey of Older Koreans (n=10,097). The results show that older adults who have more relational resources are likely to have more intention to AIP and specifically, the association is stronger at a higher awareness of services. The findings show that there is a need for a range of social engagement programs to provide more opportunities to build relational resources in later life, thus increasing the likelihood of AIP. Policy efforts are also needed to increase awareness of social services among older adults and so, they can plan their AIP with the assurance of various available supports.

